# Electrochemical Paper-Based Biosensor Devices for Rapid Detection of Biomarkers

**DOI:** 10.3390/s20040967

**Published:** 2020-02-11

**Authors:** Manuel Gutiérrez-Capitán, Antonio Baldi, César Fernández-Sánchez

**Affiliations:** 1 Instituto de Microelectrónica de Barcelona (IMB-CNM), CSIC, Campus de la UAB, 08193 Bellaterra, Barcelona, Spain; antoni.baldi@imb-cnm.csic.es; 2 CIBER de Bioingeniería, Biomateriales y Nanomedicina (CIBER-BBN), Jordi Girona 18-26, 08034 Barcelona, Spain

**Keywords:** paper-based device, electrochemical detection, biomarker analysis, paper microfluidics, point-of-care, point-of-need

## Abstract

In healthcare, new diagnostic tools that help in the diagnosis, prognosis, and monitoring of diseases rapidly and accurately are in high demand. For in-situ measurement of disease or infection biomarkers, point-of-care devices provide a dramatic speed advantage over conventional techniques, thus aiding clinicians in decision-making. During the last decade, paper-based analytical devices, combining paper substrates and electrochemical detection components, have emerged as important point-of-need diagnostic tools. This review highlights significant works on this topic over the last five years, from 2015 to 2019. The most relevant articles published in 2018 and 2019 are examined in detail, focusing on device fabrication techniques and materials applied to the production of paper fluidic and electrochemical cell architectures as well as on the final device assembly. Two main approaches were identified, that are, on one hand, those ones where the fabrication of the electrochemical cell is done on the paper substrate, where the fluidic structures are also defined, and, on the other hand, the fabrication of those ones where the electrochemical cell and liquid-driving paper component are defined on different substrates and then heterogeneously assembled. The main limitations of the current technologies are outlined and an outlook on the current technology status and future prospects is given.

## 1. Introduction

For the proper healthcare of people, rapid, accurate, and minimally invasive diagnostic tools are in high demand that enable assessing the onset and/or monitoring of diseases by detecting specific disease biomarkers. Current routine off-site analyses take several days, provoking a delay in therapeutic decisions and sometimes assuming risks due to the precautionary prescription of medicines likely showing a wide range of side effects. For in-situ analyses, the so-called point-of-care (POC) devices group a wide range of diagnostic tools, exhibiting a dramatic speed advantage over conventional techniques, allowing the early detection of biomarkers, and thus facilitating proactive disease treatment and consequently avoiding disease progression to more serious states. Not only disease, but overall patient status, monitoring could be carried out with POC devices, these being part of the so-called personalized medicine. Paper-based analytical devices, combining a paper substrate and electrochemical detection, appear to be very convenient for different healthcare scenarios. Among the many advantages of these two components, cellulose paper is flexible, biocompatible, eco-friendly, inexpensive, widely-available, light-weight, and hydrophilic. In addition, its surface can easily be chemically- and physically-modified, cut, folded, and/or stacked. One of the most important characteristics of the paper is its porosity, which allows the solution to flow via capillary action without the need for external pumping sources. Electrochemical detection approaches show well-known advantages, such as their inherent small size, low cost, low power consumption, portability, high selectivity and sensitivity, as well as the availability of a large number measuring techniques, which can be adapted to different analytical detection schemes.

**Figure 1:**
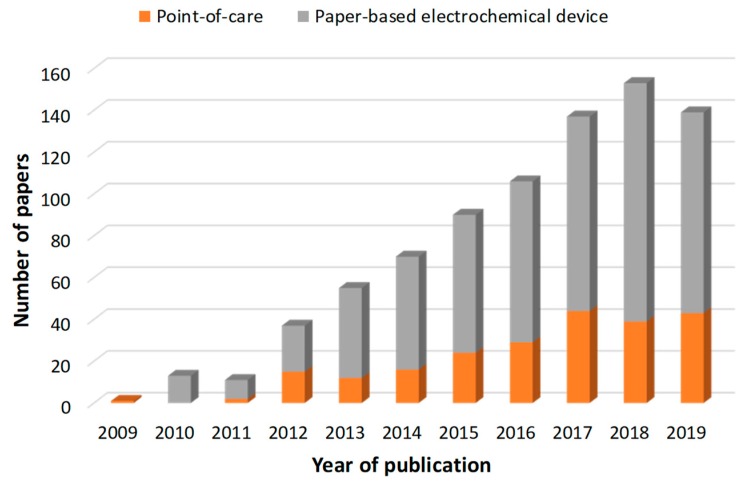
Annual trend in the number of publications for “paper-based electrochemical device” (grey) and “point-of-care” applications (orange) from 2009 to 2019. Database: Web of Science Core Collection.

The publication of research works in this field has experienced an almost exponential progression and it seems to have leveled off during the last year. A bibliographic search on the Web of Science portal, using the topic “paper-based electrochemical device” reported 812 papers since 2009, which was the year of publication of the pioneering work by C. S. Henry et al. [1], showing the potential of coupling electrochemical detection and paper-based microfluidic approaches for the multiplex detection of glucose, lactate, and uric acid in biological samples. Of those 812 works, 225 described “point-of-care” applications distributed in time, as can be seen in the bar chart (Figure 1). Among them, around 70% were clearly paper-based electrochemical devices for biomedical point-of-care applications.

Such a number of works have also resulted in many review articles published during the last five years. Good examples are one that focused on nanoparticle-based lateral flow biosensors that was published in 2015 [2] and three mainly focused on biomedical diagnostic applications that were published in 2017 [3,4] and 2018 [5]. In these reviews, different detection techniques were discussed, mainly optical transduction approaches. A more specific review was published in 2018 that addressed the field of integrated electrochemical biosensors fabricated on different flexible materials, including paper-based devices [6]. Although two other works were published in 2018 [7,8], it has been in 2019 that the number of reviews dedicated to paper-based devices for point-of-care applications has risen [9,10,11,12,13,14,15,16,17], with three more works focused on devices with electrochemical detection [18,19,20].

This review highlights significant works on the “paper-based electrochemical devices” topic over the last five years, from 2015 to 2019. The most relevant articles published in 2018 and 2019 were examined in detail. A clear focus on the technologies behind the fabrication of both the paper microfluidics and electrochemical cell components was done. The applied materials, material functionalization/modification processes, as well as electrochemical detection principles were briefly described, too. Two main sections were defined on the basis of the different component integration strategies, that is devices with the electrochemical transducers and the fluidic structures fabricated onto a single paper substrate and devices where the electrodes are fabricated onto a separated substrate and then assembled with the paper fluidic component.

## 2. Paper-Based Analytical Devices (PADs) with Integrated Electrochemical Cell

Electrodes are fabricated onto paper substrates, mainly by screen printing techniques. Electrochemical cells are defined and the surface of the working electrodes is usually modified to make it selective to the target biomarker. Devices are mostly manufactured by wax-printing processes to pattern the different areas on the paper, though a range of different technologies could be applied in this regard [9].

Analyses are usually carried out by drop-casting the required solutions, so the sample and/or reagents are added directly in the hydrophilic detection area where the electrochemical cell is defined. Regarding the microfluidic part, many of the reviewed works are based on the “origami” strategy, which permits the fabrication of 3D devices onto a single flat paper substrate by one patterning step. Then, the assembly is carried out by simply folding the paper by hand. Since 2011, when Richard M. Crooks and coworkers presented the principles of origami [21], it has become very popular due to some key advantages. That is, the single fabrication step accelerates the device production regardless of the device architecture complexity and the fabrication costs are very low since no specific tools or special alignment techniques are required.

**Figure 2:**
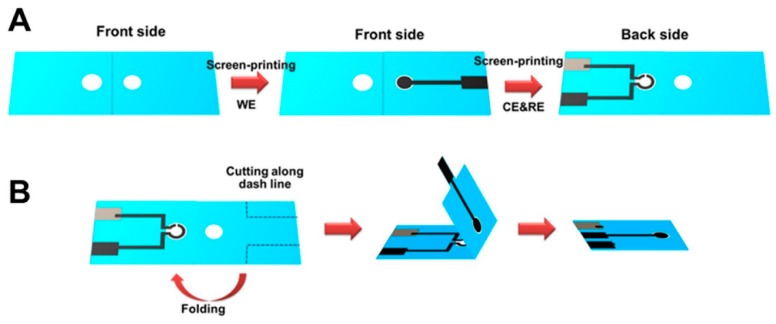
(**A**) Fabrication procedure of paper-based electrochemical device for human IFN-γ detection and (**B**) origami folding sequence. Reprinted from [22] with permission from Elsevier.

A disposable, label-free impedance immunosensor for human interferon gamma (IFN-γ) detection was developed by using Whatman filter paper grade No. 1 as substrate. The wax-patterned device was separated into two tabs, one to screen print the working electrode (WE) and the other to screen print the reference (RE) and counter electrodes (CE). This design reduced the reagent and sample volumes and prevented the contamination of the counter and reference electrodes during the modification of the working electrode. To increase the sensitivity of the immunosensor, graphene ink was used for the fabrication of the working electrode (3 mm diameter), which was then modified with polyaniline in order to covalently immobilize human IFN-γ monoclonal antibodies. Once functionalized, the two tabs of the device were folded over one another, in a one-step origami sequence, and thus the complete electrochemical cell was arranged [22] (Figure 2).

Likewise, a disposable strip for user-friendly glutathione detection in blood was reported. The device was fabricated onto filter paper (67 g/m^2^), where the waxed hydrophobic structure confined the solution in the electrochemical cell area, avoiding its diffusion towards the electric contacts. The WE (4 mm diameter) and CE were manually screen-printed by using graphite ink modified with 5% (w/w) Prussian Blue/carbon black powder. Then, cystamine was deposited by drop-casting onto the WE. The detection is based on the thiol-disulfide exchange reaction between this pre-loaded cystamine and the glutathione, which was previously released through a process of blood lysis. This reaction produces cysteamine, a compound easily oxidizable thanks to the electrocatalytic properties of Prussian Blue incorporated in the WE [23]. Moreover, a so-called lab-on-paper device was fabricated for the detection of the breast cancer MCF-7 cell line by using Whatman chromatography paper grade No. 2 as the substrate. This device consisted of three wax-printed patterned separated areas that eventually folded in an origami-like configuration. Channel and reference areas were produced together with one detection area that defined an 8.00-mm diameter hydrophilic working zone where a carbon WE was screen-printed. The reference area included a carbon CE and an Ag/AgCl RE also screen-printed onto a defined 8.00-mm diameter hydrophilic zone. The WE was modified with three-dimensional reduced graphene oxide (3D-rGO) onto which Au nanoparticles were subsequently synthetized by a chemical reduction process. Finally, MCF-7 cell-specific aptamer H1 was deposited on the formed Au@3D-rGO. The different patterned areas were then folded in a two-step origami sequence so that the whole screen-printed, three-electrode electrochemical cell was easily connected once the paper unit was filled with solution [24].

In a different approach, finger-type silver-carbon electrode pairs were embedded in a paper substrate and applied to the impedimetric detection of α-fetoprotein tumor biomarker in human serum. The devices were prepared from a lower flexible sheet of plastic (3M, Italy) and an upper layer of aldehyde-modified cellulose-paper substrate (Whatman chromatography paper). The finger type electrodes were screen-printed with different conductive materials including silver ink, graphene paste, and silver-graphene nanocomposite (silver-20 wt% graphene paste) using a stencil mask. Diphenylalanine nanotubes were deposited on the paper in order to incorporate aldehyde groups and facilitate the covalent immobilization of antibodies to the target analyte [25].

**Figure 3:**
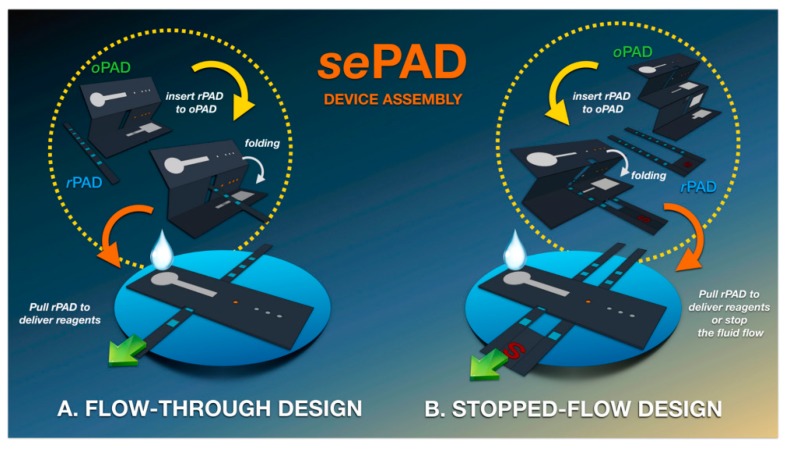
Schematic illustration of the sequential paper analytical device (sePAD) components and device assembly using (**A**) flow-through design and (**B**) stopped-flow design. Reprinted from [26] with permission from American Chemical Society (ACS).

A 3D sequential fluid delivery platform on a microfluidic paper-based device (sePAD) was fabricated. It is capable of storing and transporting reagents sequentially to the detection channel without the need for external power, thus eliminating the multiple-step reagent manipulation inherent to complex bioassays. The device is comprised of two components, that is an origami folding paper (oPAD) and a movable reagent-stored pad (rPAD) with two different configurations: the flow-through architecture, developed for continuous flow electrochemical measurements, such as chronoamperometry, and the stop-flow architecture, developed for non-convective electrochemical measurements, such as voltammetry. In both cases, a wax-printing technique was used to pattern Whatman grade 1 chromatography paper. Next, hollow channels were cut using a razor blade. Three electrodes were then screen-printed using carbon/graphene paste and Ag/AgCl ink at the back of the detection zone of the oPAD. The rPAD was placed in the folding paper of the oPAD, and the assembled device was sandwiched between two acrylic plates and tightened with binder clips (Figure 3). This 3D capillary-driven device was used for the determination of ascorbic acid and serotonin and as an impedimetric label-free immunosensor for α-fetoprotein [26] (Figure 3).

Wax printing is a fast and simple fabrication technique for patterning the hydrophobic areas on paper. However, its resolution is limited by the temperature post-treatment required for fully blocking the paper substrate due to lateral diffusion of the melted wax. Moreover, the commercial availability of wax printers has been discontinued, which may hamper the potential widespread use of this approach in the future. Thus, patterning of hydrophobic areas on paper was carried out by other alternative approaches. Alkyl-ketene dimer (AKD)-inkjet printing was also used to define hydrophobic barriers. This technique rapidly fabricates devices on a large scale with better resolution than wax printing, but with the requirement of more expensive inkjet printer instrumentation. When greater precision and control are required, a previous optimization process has to be performed for the precise adjustment of ink droplet volume, ejection speed, and spacing. In this context, a PAD for serotonin determination was reported [27]. Two 8-mm diameter circled hydrophilic areas were defined on Whatman filter paper grade No. 1 substrates using AKD. A three-electrode electrochemical cell (WE, CE and RE) was screen-printed onto one these areas, using a custom-made carbon ink comprising graphite powder, carbon nanotubes (CNTs), and mineral oil. The WE was modified with Fe_3_O_4_@Au@SiO_2_ nanoparticles coated with molecularly imprinted polymer (MIP), selective to serotonin. Once functionalized, the device was folded, in a one-step origami sequence, in such a way that both the hydrophilic circled areas matched. The one not including the electrochemical cell minimized the direct contact between the fabricated electrodes and the sample, accommodating larger sample volumes and ensuring good sample impregnation onto the fabricated electrodes.

Apart from the origami strategy followed in the previously described works, there are other reported approaches where the electrochemical cell and the microfluidic components are fabricated in separated paper substrates. This implies the implementation of alignment strategies to ensure a good and correct fitting between the different paper components. One good example is the fabrication of a label-free aptasensor device on 10.5 × 35.0-mm^2^ pieces of Whatman chromatography paper grade No. 1 for the simultaneous multiplexed detection of two cancer biomarkers, namely carcinoembryonic antigen (CEA) and neuronspecific enolase (NSE), in clinical samples. The wax-printing technique was used for patterning the different paper layers. The upper layer included the sample inlet and a cropped cellulose filter hole. In the next layer, two circle detection zones were designed, where the respective CE and RE were screen-printed using conductive carbon ink and Ag/AgCl ink, respectively. The third layer of paper contained a microchannel to flow the sample to the two different detection zones, one for the detection of CEA and the other for NSE. In the bottom piece of the paper, the two carbon WEs were screen-printed. In order to promote the electron transfer and to immobilize the respective aptamers, these WEs were modified, one with amino functional graphene (NG)-thionin (THI)-gold nanoparticles (AuNPs) nanocomposite and the other with Prussian Blue (PB)-poly(3,4-ethylenedioxythiophene) (PEDOT)-AuNPs nanocomposite. Finally, the four paper components were assembled using double-sided tape [28]. The same device was also used for point-of-care testing of 17β-estradiol in clinical serum samples, but using multi-walled carbon nanotubes-THI-AuNPs nanocomposite onto the WE and immobilizing a specific antibody [29].

**Figure 4:**
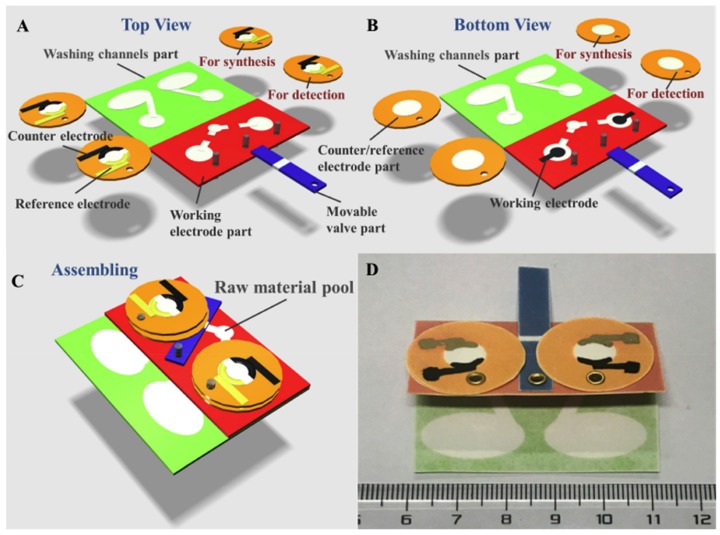
(**A**) Top view and (**B**) bottom view of the different components integrated in the device. (**C**) Assembled device. (**D**) Photograph of the top view and size scale of the device. Reprinted from [30] with permission from Elsevier.

Another paper used the layered configuration for the detection of CEA in human serum samples. A molecularly imprinted polymer (MIP)—electrosynthesized polymer in the presence of the target analyte—was electrosynthesized on a structured area of the device and used as the specific receptor for this target analytes. Likewise, a non-imprinted polymer (NIP–electrosynthesized polymer without the target analyte) was produced in the same fashion as the MIP in a separated but identical area of the device. These two separate but identical structured areas comprised three substrates of Whatman chromatography paper grade No. 1, patterned by wax printing, which accommodated: a carbon WE (50 × 25 mm^2^), Ag/AgCl CE/RE (24 mm in diameter), and a channel architecture (40 × 50 mm^2^). The electrodes were produced by manually brushing the corresponding inks on the paper substrate. A movable valve was defined in an extra paper component for enabling continuous and convenient delivery of the different liquids required for the MIP/NIP syntheses and further electrochemical analysis [30] (Figure 4).

Considering the technology presented here to be application-driven, some recently developed paper-based platforms could be customized depending on the target biomarker. For example, a conductive platform was generated onto ivory paper sheet (15 × 5 mm^2^) by dipping them in a graphene oxide (GO) dispersion and subsequently reducing the GO with hydrazine. Then, cysteine-capped gold nanoparticles (Cys-AuNPs) were electrophoretically deposited onto the reduced GO (RGO) paper previously generated. The cysteine residues were used to covalently immobilize, through the carbodiimide reaction, monoclonal antibodies. Interleukin-8 (IL-8), a cancer biomarker, was used as model target in this study [31]. Also, a PAD platform was fabricated with a hollow 3D analyte reservoir that enabled the use of a more uniform screen-printed electrode top surface as the electroactive sensing area. As a proof of concept, the device was implemented for pH, glucose, and dopamine detection, which required potentiometric, amperometric, and voltammetric readout detection schemes, respectively [32]. A new platform for the electrochemical detection of single- and double-stranded DNA was developed using paper-based screen-printed electrodes. The optimized device was used to analyze different target DNA strands in undiluted serum [33]. A novel form of paper-based biosensor was reported with hierarchical assembled nanomaterials and metalorganic framework-enhanced bioprobes for the simultaneous electrochemical detection of microRNAs. In this case, the platform was used to determine microRNA-141 and microRNA-21 in human serum samples [34]. Another 3D paper-based platform, created via combining thin adhesive films and paper folding, was recently published for simultaneously running assays in different layers. Its feasibility was demonstrated using glucose as the target analyte [35]. Finally, an H_2_O_2_-controlled fluid switch-mediated paper-based biochip for multiplexed and quantitative analysis was recently developed and applied to the detection of MCF-7 and K562 cells models [36]. The most important analytical characteristics of these devices are summarized in Table 1.

Other relevant works with the electrochemical cell integrated in different PADs published between 2015 and 2019 are summarized in Table 2.

The analytical approaches presented in this section are quite appealing from a fabrication point-of-view because they combine different technologies and means for integrating all the components of the device on paper substrates that, in most cases, could be inherently aligned because they rely on origami-based folding steps to produce the final device architecture. Moreover, they can be produced at a very low cost without the requirement of expensive instrumentation. However, the process of screen-printing the electrodes on the paper substrate is not that straightforward considering the porosity of the material and its limited mechanical robustness. Moreover, most of the reported approaches rely on several manual steps for device fabrication and performance when movable parts are included. This should be avoided when aiming to produce prototypes with potential application in real scenarios.

## 3. Paper-Based Analytical Devices (PADs) with Non-Integrated Electrochemical Cell

Electrodes fabricated on substrates other than paper, using different techniques are outlined in this section. Here, paper is just applied to develop the fluidic component of the final device.

**Figure 5:**
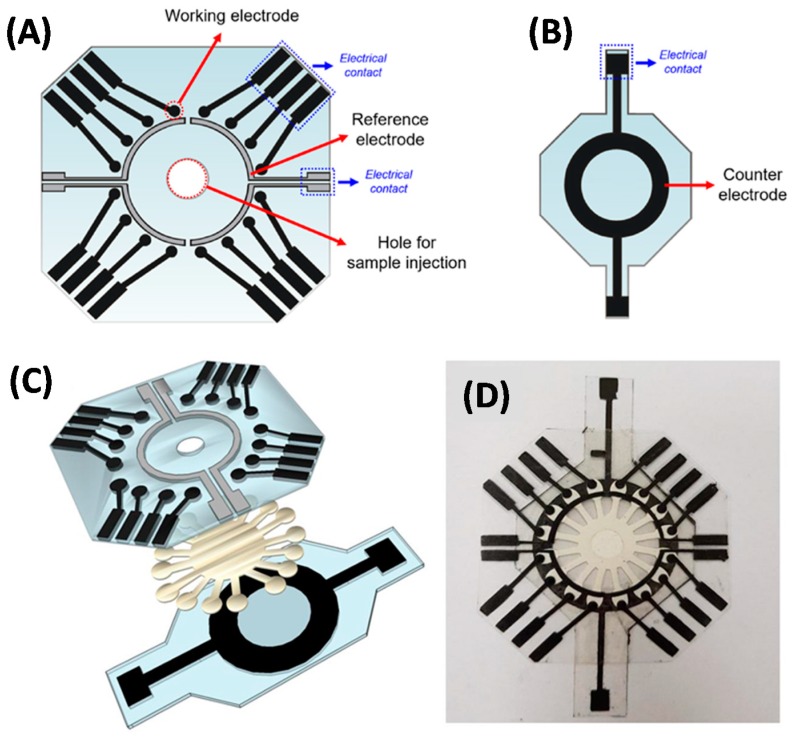
(**A**) Design of the WEs/REs and (**B**) CE layers. (**C**) Steps for device layer assembly. (**D**) Photography of assembled multiplexed PAD. Reprinted from [53] with permission from Elsevier.

One very interesting recent work by O. Fatibello-Filho and co-workers is focused on the development of a disposable electrochemical PAD comprising 16 independent microfluidic channels coupled to the same number of individually addressable electrochemical cells and is applied to the multiplex glucose determination in human urine samples. The microfluidic paper component includes the 16-channels radially distributed around the sample addition area and thus separated 22.5° from each other. This distribution provides a radial elution of the sample, with homogeneous flow of the solutes into the 16 equidistant sensing points. Here, a physical patterning process of Whatman filter paper grade No. 1 was carried out using a craft cutter printer (see Figure 5). In fact, the major advantage of this work was the lack of wax printing to produce the 16-channel paper component, which reduces the cost of operation. Electrodes were fabricated by a stencil printed approach using a vinyl adhesive mask containing the pattern. The mask was attached to a polyester substrate and conductive carbon ink was applied by screen-printing. For the fabrication of the REs, an additional step, consisting of deposition of Ag/AgCl ink layer onto the previous carbon ink, was performed. The WEs and the REs were made in the same polyester sheet to keep the distance between them constant. Each of the 16 WEs had independent electrical contacts, while each set of four WEs had one RE, resulting in four REs included in the overall device (Figure 5A). In order to perform the glucose detection, the WEs were then modified with a film of chitosan, containing carbon black and glucose oxidase, together with ferrocene-carboxylic acid as the redox mediator. As can be seen in Figure 5B, a circular CE was fabricated in a separated sheet and arranged so that it was in contact with all 16 electrochemical cells and kept at a set distance with the WEs and REs. Finally, the assembly and sealing of the integrated PAD was performed using double-sided adhesive (Figure 5C,D) [53].

The same group developed a simpler disposable PAD, consisting of two electrochemical sensors, for the simultaneous determination of uric acid and creatinine in human urine samples. In this case, the filter paper layout just consists of a sample injection spot and a common detection area, also cut using a home cutter printer. The electrode fabrication technology is the same as above. Selective receptors for both target analytes were included with one WE modified with graphene quantum dots for direct oxidation of uric acid and the other one modified with graphene quantum dots, creatininase enzyme, and hexaammineruthenium (III) chloride as the redox mediator for the electrocatalysis of creatinine [54].

Other good example is a disposable PAD developed for the label-free detection of C-reactive protein in certified human serum. The PAD consists of three parts, including the paper part, the screen-printed electrodes, and double-sided tape. The electrodes were constructed onto a PVC substrate, where the RE and conductive pads were screen-printed with Ag/AgCl ink. Then, the carbon ink was printed for producing both the WE and CE. The WE (4 mm in diameter) was modified by electrodeposition of gold nanoparticles, where a self-assembled monolayer of thiol-terminated poly(2-methacryloyloxyethyl phosphorylcholine) (PMPC-SH) was formed. The paper part was fabricated by wax printing using Whatman filter paper grade No. 1 as substrate. This includes three different parts: the middle zone was defined for the attachment of the screen-printed electrodes, whereas one flap area was used for the storage of Ca^2+^ and incubation of the sample, and the other flap was used for the storage of the K_3_Fe(CN)_6_ redox probe and analysis of the sample. Finally, a 20 × 20 mm^2^ double-sided tape was cut and a circled area punctured to be placed above the electrochemical cell. The detection was based on the specific binding between the phospholipid structure of PMPC-SH onto the surface of the WE with C-reactive protein, which produced a decrease in the voltammetric response of the redox probe [55].

**Figure 6:**
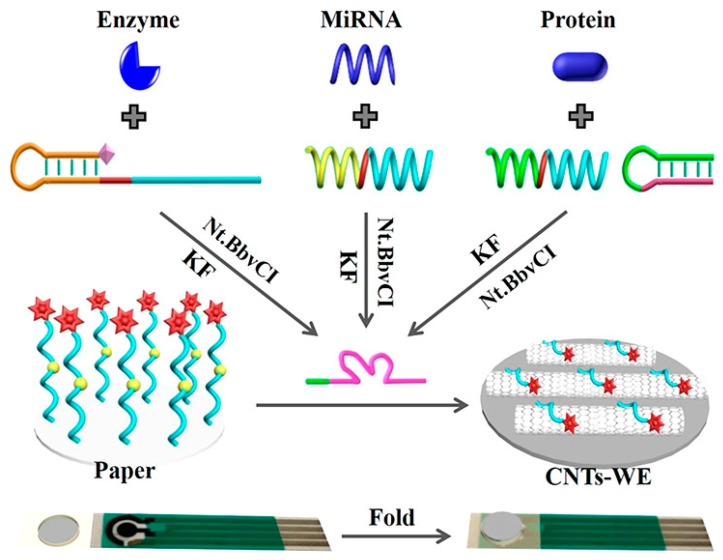
Scheme of the use of commercial screen-printed electrodes combined with a functionalized paper. MiRNA: microRNA; KF: Klenow fragment; Nt.BbvCI: nicking endonuclease. Reprinted from [56] with permission from American Chemical Society (ACS).

Another simpler and effective strategy is the use of commercial screen-printed electrodes combined with a functionalized paper, as in [56]. The working electrode was just modified with CNTs, in order to increase the active area and amplify the electrochemical signal. The paper component, consisting of 5 mm diameter circular pieces, was produced on Whatman cellulose chromatography paper by a CO_2_ laser cutting machine. Then, the circular paper was treated with NaIO_4_ for forming aldehyde groups to immobilize the ferrocene-labeled DNA (Fc-DNA) strand through a Schiff alkali reaction. For the assembly, the obtained Fc-DNA-modified paper was combined with a commercial screen-printed electrode by sticking them onto a soft plastic slide and folding by using a double-sided adhesive tape, so that the paper was just on the electrochemical cell and performing the analysis by drop-casting. The assays relied on the target-induced synthesis of Mg^2+^-dependent DNAzyme for catalyzing the cleavage of Fc-DNA from paper, which had been proved by using microRNA recognition probe for miR-21, a biomarker for cardiovascular disease and cancer, a phosphorylated hairpin probe for alkaline phosphatase enzyme, and a DNA aptamer for CEA, respectively (Figure 6).

Other relevant works that fit within this section and that were published between 2015 and 2019 describe a disposable PAD immunosensor based on screen-printed electrodes and a paper microfluidic component fabricated by photolithography for the detection of α-fetoprotein biomarker in human serum samples [57], a PAD based on stencil-printed electrodes onto plastic transparency sheets and an origami paper microfluidic approach for the detection of the kidney disease biomarker Trefoil Factor 3 (TFF3) in human urine samples [58], and a label-free approach for the detection of virus particles, such as West Nile Virus, based on a microfluidic PAD with integrated microwire gold electrodes [59].

The most important analytical characteristics of these devices are summarized in Table 3.

Devices included in this section stand out for their ease of fabrication and huge flexibility considering that the electrochemical transducers are produced in a separate process to that of the paper fluidic component. Moreover, the fluidic features are only dependent on the selected paper, taking into account that just a patterning process for producing the desired fluidic structures is required. However, alignment and pressure conditions between the two components should be strictly controlled to not limit the performance of the device and its fabrication and analytical reproducibility.

## 4. Conclusions and Outlook

While there have been a myriad of electrochemical PADs with potential application in biomedicine, there is still some important pending issues that should be tackled before they can be really competitive and enter the POC market.

Since Whitesides and co-workers’ pioneering work on paper microfluidics in 2007 [60], there has been a huge progress in the development of patterned paper architectures using a bunch of alternative fabrication technologies and the integration of different detection strategies in order to get a quantitative analytical response. However, in spite of the significant inherent features of the paper material, such as availability, very low cost, simple and secure manipulation and disposability, as well as capillary flow properties, amenability for reagent integration, and easy patterning, it suffers from important limitations in terms of reproducibility, long-term stability, and multiplexing capabilities. Henry and co-workers, in one of their last reviews on this topic [15], pointed out that, considering the huge effort that the research community is putting to overcome these hurdles, effective engineering solutions will be reported in the near future.

Electrochemical detection approaches have already shown superior performance when coupled to paper fluidic components. They are versatile detection approaches that can be easily adapted in terms of electrode materials and electrochemical cell configuration as well as electrochemical techniques and required instrumentation. Nevertheless, there is still room for improvement regarding electrode robustness and reproducibility, mainly when electrochemical cells are implemented on paper substrates. Screen printing is mainly being used for this process and, although it is a very cost-effective parallel fabrication technique, the fabricated electrodes often suffer from limited sensitivity and reproducibility. Likewise, electrode architectures cannot be downsized below certain dimensions. In this context, the integration of robust electrochemical cells fabricated on different substrates by other techniques, such as material evaporation - photolithographic approaches, could be an important alternative to improve the overall device performance. Device size could be further reduced, if necessary, and more highly reproducible results could also be achieved. Although it is a more expensive alternative, these techniques could be applied for producing electrochemical transducers that could be reused for consecutive measurements, so that the cost per analysis could be kept low. Electrochemical detection approaches require low-powered instrumentation that can be compact and custom made for a particular application. There are different technologies already on the market that fulfill these requirements and that should be seriously considered. However, most of the reported devices neglect this and have been characterized by bulky commercial bench-top equipment. This is important for device deployment and the final price and should be clearly addressed during device development.

The application of electrochemical paper-based biosensor approaches for point-of-care applications will not be a reality if the analyses involve several manual steps or require complex architectures that would hamper the eventual device mass production. Devices should require very little user manipulation and result in very low cost-per-analysis if these are to be widespread in screening programs, for home self-testing, or bedside testing. In this context, a sample-to-answer analytical tool will be ideal and so integrated sample pretreatment approaches should be fully implemented. This is difficult when working with biological samples and a large effort is still required in this direction.

The scientific community is well aware of the market pull for biomedical solutions that help detect biomarkers that carry out disease diagnosis and prognosis more efficiently. We believe the combination of paper fluidics and electrochemical transduction will result in devices of highly added value to be at the forefront for market exploitation.

## Figures and Tables

**Table 1:** Analytical features of the described PADs with integrated electrochemical cell.

Biomarker ^1^	Technique ^2^	Response Range	Sensitivity ^3^	Limit of Detection	Ref.
IFN-γ	EIS	5–1000 pg/mL	33.2 kΩ/dec	3.4 pg/mL	[22]
Glutathione	Amp.	0.25–10 mM	0.102 ± 0.005 µA/mM	0.06 mM	[23]
MCF-7 line	DPV	50–10^7^ cells/mL	−6.8 µA/dec	20 cells/mL	[24]
Serotonin	LSV	0.01–1000 µM	0.008 ± 0.005 µA/µM	0.002 µM	[27]
α-fetoprotein	EIS	1–10^4^ ng/mL	-	10 ng/mL	[25]
Ascorbic acid	Amp.	0.15–0.8 mM	7.8 µC/mM	0.093 mM	[26]
Serotonin	DPV	1–20 mM	0.16 µA/mM	0.15 mM	[26]
α-fetoprotein	EIS	10–100 ng/mL	10 kΩ/dec	0.63 ng/mL	[26]
CEA	DPV	0.01–500 ng/mL	−2.8 µA/dec	0.002 ng/mL	[28]
NSE	DPV	0.05–500 ng/mL	−1.4 µA/dec	0.01 ng/mL	[28]
17β-estradiol	DPV	0.01–100 ng/mL	−1.8 µA/dec	0.01 ng/mL	[29]
CEA	DPV	1.0–500.0 ng/mL	19.3 µA/dec	0.32 ng/mL	[30]
IL-8	Chronoamp.	1–9 pg/mL	−1.64 µA mL/pg	0.59 pg/mL	[31]
pH	Potent.	2–12 pH	−45 mV/pH	-	[32]
Glucose	Chronoamp.	5–17.5 mM	0.34 µA/mM	-	[32]
Dopamine	CV	0.01–5 mM	-	-	[32]
DNA targets	SWV	-	-	3 to 7 nM	[33]
miR-141	SWV	1 fM–1 nM	-	0.1 fM	[34]
miR-21	SWV	1 fM–1 nM	-	0.1 fM	[34]
Glucose	Chronoamp.	1–40 mM	−0.091 µA/mM	0.32 mM	[35]
MCF-7 cells	DPV	150–10^7^ cells/mL	0.12/dec ^4^	117 cells/mL	[36]
K562 cells	DPV	220–7 × 10^6^ cells/mL	0.13/dec ^4^	140 cells/mL	[36]

^1^ IFN-γ: human interferon gamma; CEA: carcinoembryonic antigen; NSE: neuronspecific enolase; IL-8: interleukin-8; miR-141: microRNA-141; miR-21: microRNA-21. ^2^ EIS: electrochemical impedance spectroscopy; Amp.: amperometry; DPV: differential pulse voltammetry; LSV: linear sweep voltammetry; Chronoamp.: chronoamperometry; Potent.: potentiometry; CV: cyclic voltammetry; SWV: square wave voltammetry. ^3^ dec means log of the biomarker concentration. ^4^ Non-dimensional sensitivity values related to the ratio between two different analytical signals.

**Table 2:** Other relevant PADs with integrated electrochemical cell published between 2015 and 2019.

Device	Biomarker	Type of Sample	Technique ^1^	Response Range	Sensitivity ^2^	Limit of Detection	Ref.
Peptide nucleic acid biosensor	Human papillomavirus	PCR-amplified DNA from SiHa cell line	SWV	10–200 nM	0.004 µA/nM	2.3 nM	[37]
3D “pop-up” with commercial glucometer	Beta-hydroxybutyrate	Whole blood	Chronoamp.	0.1–6.0 mM	-	0.3 mM	[38]
Label-free immunosensor	Biotin-avidin interaction	Standard solution	Chronoamp.	Up to 500 ng/mL	0.33 µA mL/ng	25 ng/mL	[39]
Wireless potentiometric platform	Glucose	Blood	Potent.	0.3–3 mM	-96±5 mV/dec	0.1 mM	[40]
Non-enzymatic sensor	Creatinine	Human blood serum	Chronoamp.	0.01–2.0 mM	28 µA/cm^2^ mM	0.22 µM	[41]
Label-free immunosensor	Cancer antigen 125	Quality control serum	DPV	0.1–200 U/mL	−0.37 µA mL/U	0.01 U/mL	[42]
Label-free aptasensor	Prostate specific antigen	Clinical serum	DPV	0.05–200 ng/mL	−2.0 µA/dec	10 pg/mL	[43]
Voltammetric sensor	3-nitrotyrosine	Standard solution	SWV	0.5 µM–1 mM	-	49.2 nM	[44]
Reagent-free 3D printing device	Butyrylcholinesterase activity	Serum	Chronoamp.	1–12 IU/mL	0.23 ± 0.01 µA mL/UI	0.1 IU/mL	[45]
Disposable non-enzymatic sensor	Glucose	Human serum	Chronoamp.	0.01–1.3 mM	0.016 µA/µM	0.64 µM	[46]
Enzymatic biosensor with pre-loaded (bio)reagents	Glucose	Whole human blood	Chronoamp.	Up to 25 mM	-	-	[47]
Label-free aptasensor	17β-estradiol	Clinical serum	DPV	0.01–500 ng/mL	−2.36 µA/dec	5 pg/mL	[48]
Label-free immunosensor	Cortisol	Human saliva	EIS	3 pg/mL–10 µg/mL	50 Ω mL/pg	3 pg/mL	[49]
Enzymatic biosensor with pre-loaded (bio)reagents	Glucose	Whole human blood	Chronoamp.	1–12 mM	0.9474 µA/mM	0.05 mM	[50]
Enzymatic biosensor with CeO_2_ catalyst	miR-21	Diluted human serum	DPV	1.0–1000 fM	−6.22 µA/dec	0.434 fM	[51]
Enzymatic biosensor with pre-loaded (bio)reagents	Glucose	Spiked human serum	Chronoamp.	1–20 mM	-	1 mM	[52]

^1^ SWV: square wave voltammetry; Chronoamp.: chronoamperometry; Potent.: potentiometry; DPV: differential pulse voltammetry; EIS: electrochemical impedance spectroscopy. ^2^ dec means log of the biomarker concentration.

**Table 3:** Analytical features of the PADs with non-integrated electrochemical cell.

Biomarker	Technique ^2^	Response Range	Sensitivity ^3^	Limit of Detection	Ref.
Glucose	Chronoamp.	0.1–40 mM	-	0.03 mM	[53]
Uric acid	SWV	0.010–3.0 µM	0.08 ± 0.0024 µA/µM	8.4 nM	[54]
Creatinine	SWV	0.010–3.0 µM	0.30 ± 0.0057 µA/µM	3.7 nM	[54]
C-reactive protein	DPV	5–5000 ng/mL	5.51 µA/dec	1.55 ng/mL	[55]
miR-21	DPV	1 fM–1 µM	5.33 nA/dec	-	[56]
Alpha-fetoprotein	SWV	0.01–100 ng/mL	−12.698 µA/dec	0.005 ng/mL	[57]
TFF3 ^1^	ASV	0.0125–3 µg/mL	-	0.0125 µg/mL	[58]
West Nile virus	EIS	Up to 10^6^ particles/mL	-	2000 particles/mL	[59]

^1^ TFF3: trefoil factor 3. ^2^ Chronoamp.: chronoamperometry; SWV: square wave voltammetry; DPV: differential pulse voltammetry; ASV: anodic stripping voltammetry; EIS: electrochemical impedance spectroscopy. ^3^ dec means log of the biomarker concentration.
